# Awareness of the public charge, confidence in knowledge, and the use of public healthcare programs among Mexican-origin Oregon Latino/as

**DOI:** 10.1186/s12939-023-02027-w

**Published:** 2023-10-10

**Authors:** Edlyn Wolwowicz-Lopez, Emily Boniface, Sara Díaz-Anaya, Yareli Cornejo-Torres, Blair G. Darney

**Affiliations:** 1 Oregon Health and Science University, Portland, OR USA; 2 Portland State University, Portland, OR USA; 3 OHSU-PSU School of Public Health, 506 SW Mill St, Portland, OR 97201 USA; 4 National Institute of Public Health (INSP), Center for Population Health (CISP), Cuernavaca, Morelos, Mexico

**Keywords:** Public charge rule, Latino/a, Insurance, State healthcare programs

## Abstract

**Objective:**

We describe awareness about the modified “public charge” rule among Oregon’s Mexican-origin Latino/a population and whether concerns about the rule influenced disenrollment from state-funded programs, which do not fall under the public charge.

**Methods:**

We conducted a cross-sectional survey of adults (ages 18–59) recruited at the Mexican consulate and living in the state of Oregon. Our outcomes were awareness (of the public charge, source of knowledge, and confidence in knowledge of the public charge) and disenrolling self or family members from state-funded public healthcare programs due to concerns about the rule. We described outcomes and used logistic regression and calculated adjusted probabilities to identify factors associated with awareness of the public charge.

**Results:**

Of 498 Latino/a respondents, 48% reported awareness of the public charge. Among those who knew about the public charge, 14.6% had disenrolled themselves or family members from public healthcare programs and 12.1% were hesitant to seek care due to concerns about the public charge. Younger respondents had a lower adjusted probability of awareness of the public charge (18–24 years: 15.6% (95% CI 3.1–28.2); 30–39 years 54.9% (95% CI 47.7–62.0). Higher education was associated with a higher adjusted probability of awareness of the public charge; ability to speak English was not associated with awareness of the public charge.

**Conclusion:**

Our study reveals limited awareness about the public charge among Mexican-origin Oregon Latino/as. Outreach and advocacy are essential to ensure Latino/as know their rights to access available state-funded healthcare programs.

## Introduction

The public charge is a set of rules associated with the Immigration Nationality Act that outlines a list of federal public benefits and defines immigrants who have accessed these benefits as likely to become a “economic burden” or a “public charge” [1–3]. Being classified as a “public charge” can lead to denial of applications for changes in immigration status (e.g. naturalization or long-term permanent resident [LTPR or “green card”] applications) [1]. In 2019, the Trump administration modified the public charge rule to add federally funded Medicaid health insurance and additional income assistance programs to the list of programs that classify someone as a “public charge” [1, 2, 4].

Concern about the public charge is widespread among immigrant and US-born communities, and mixed-status families [5]. Although the public charge targets only specific immigrants (e.g. does not apply to refugees) [4], does not include any programs funded using state dollars, and enrollment in programs by children does not impact parental “public charge” status, there is clear evidence of a “chilling effect” or “spillover effect” [6] extending to immigrants in general, or even to entire racial or ethnic groups via “racialized legal status” [7] – an inferred immigration status based upon perceived race or ethnicity. For example, at the county level, child enrollment in Medicaid, SNAP and WIC declined as the share of adult non-citizens increased following the Trump changes to the public charge rule and one in seven immigrant adults reported avoiding public programs [5, 8]. In 2022, the Biden administration reversed the Trump administration changes to the public charge rule [9]; however, confusion and mistrust about the public charge rule persists [10].

In Oregon, one in ten residents is an immigrant and Mexico is the most common country of origin [11]. Oregon uses state funds to supplement federal Medicaid (called the Oregon Health Plan or OHP), to expand coverage for recent and undocumented minors [12, 13] and for reproductive health services for adult men and women [14, 15]. These state-funded programs support equity in access to healthcare and do not fall under the public charge rule. However, it is not known whether Oregon Latinos are disenrolling from state-funded healthcare programs due to concerns about the public charge. Understanding Oregon Latina/o awareness of the scope of the public charge and behaviors around disenrollment in state-funded programs can help with outreach efforts to support health equity in Oregon. The purpose of this study is to describe awareness of the public charge, confidence in knowledge about the public charge, and any disenrollment or avoidance of health service utilization due to the public charge among Oregon Latina/os. Additionally, we sought to identify factors associate with awareness of the public charge to inform outreach efforts.

## Methods

We conducted a cross-sectional survey using convenience sampling, in partnership with the Consulate General of Mexico in Portland, OR [16]. Recruitment occurred between April-June 2021. Participants were approached while waiting at the Consulate to process documents (e.g., passports, birth certificates) or to visit the “Ventanilla de Salud” which provides information and healthcare navigation services [17]. Our bicultural and bilingual Mexican-American research team recruited 500 men and women. Inclusion criteria were: self-identified as Latina/o, 18–59 years old, and lived in the state of Oregon. Participants provided oral consent and chose preferred language for the survey (Spanish or English). Participants could fill out the survey on their own or have it read to them by the research team, to reduce language and literacy barriers. Surveys were completed on paper, responses were then entered into a Research Electronic Data Capture (REDCap) [18] database by trained study members. Participants were given a pen to thank them for their participation and a resource sheet with up-to-date information about the public charge. This study was approved by the Oregon Health & Science University Institutional Review Board.

We developed our survey using published questions from the California Health Interview Survey [19] and the Urban Institute’s Well-being and Basic Need Survey [20] and tailored to Oregon public health programs [12–14]. We received input on the survey from staff of the Mexican Consulate, and two local non-profits focused on legal aid and the Latino community, respectively. Our survey contained 20 questions and included sociodemographics, awareness about the public charge; how confident they were in their understanding; where they learned about the public charge; awareness of health care programs in Oregon; whether participants had disenrolled themselves or a family member from specific Oregon health care programs due to the public charge; and whether they were hesitant to seek care due to the public charge. We also included an optional open-ended question: *in your words, what do you know about the “public charge” rule?* Respondents reported their understanding of the public charge rule as “not confident”, “somewhat confident”, “confident”, or “very confident”; we created a binary variable or lower (not or somewhat confident) vs. higher (confident or very confident).

We collected several sociodemographic characteristics. We included gender, age (categorized as 18–24, 25–29, 30–39, 40–49, and 50 or older), and years in the US (less than five, five-nine, ten or more, and “all of my life”). We included household size (four people or less versus five people or more), marital status, and education level. We included having a job or regular income and language spoken at home (Spanish, English, both Spanish and English, or other). Finally, we included self-rated ability to speak English (not at all, somewhat, or very well). All covariates except age were missing data, so we included categories for missing in Tables and for regression modeling.

We compared respondent sociodemographic characteristics by awareness of the public charge using Pearson’s chi-squared test and determined the proportion of respondents who had heard of health programs or services in Oregon and whether they had enrolled themselves or a family member in those programs. We tabulated the proportion of respondents who had heard of the public charge rule for each information source. We graphically assessed the percent of respondents who had heard of the public charge rule who had disenrolled themselves or a family member from a health program or who were hesitant to seek care due to concerns about the rule, overall and by their confidence in understanding the rule (lower vs. higher confidence). We developed a logistic regression model with awareness of the public charge rule as the outcome to determine what characteristics were associated with awareness of the rule. In order to improve the interpretability of the modeling results [21], we calculated the adjusted probability (average marginal effect) of having awareness of the public charge rule for each covariate in the final model. All analyses were performed in Stata version 15.1 (Stata Corp, College Station, TX).We extracted all open-ended responses about awareness of the public charge into an excel sheet; two study team members classified open-ended responses into preliminary themes, which were discussed and refined with team members to arrive at key themes.

## Results

Our final sample included 498 Latina/o respondents (two were excluded due to residence outside of Oregon). Two-thirds of our study sample was female (67.5%) (Table 1), most of our participants were 30–39 years old (36.1%), had been living for ten years or more in the U.S (69.5%), 25.7% of participants had less than a high school education, most (65.9%) spoke Spanish at home with one participant reporting speaking an indigenous language (Q’anjob’al, a Mayan language). Nearly all participants (94%) took the survey in Spanish (data not shown).

**Table 1:** Survey respondent characteristics, by whether they have heard of the “Public Charge” rule. Data are n (%)

Characteristic	No	Yes	Total	p-value
n	258 (52%)	240 (48%)	498 (100%)	
Sex				0.161
Female	164 (63.6)	172 (71.7)	336 (67.5)	
Male	92 (35.7)	68 (28.3)	160 (32.1)	
Missing	2 (0.8)	0 (0.0)	2 (0.4)	
Age (years)				< 0.001
18–24	32 (12.4)	5 (2.1)	37 (7.4)	
25–29	32 (12.4)	22 (9.2)	54 (10.8)	
30–39	79 (30.6)	101 (42.1)	180 (36.1)	
40–49	75 (29.1)	83 (34.6)	158 (31.7)	
≥ 50	40 (15.5)	29 (12.1)	69 (13.9)	
Years in the United States				0.028
< 5	30 (11.6)	15 (6.3)	45 (9.0)	
5–9	30 (11.6)	22 (9.2)	52 (10.4)	
≥ 10	163 (63.2)	183 (76.3)	346 (69.5)	
All my life	22 (8.5)	13 (5.4)	35 (7.0)	
Missing	13 (5.0)	7 (2.9)	20 (4.0)	
Number of people in household				0.040
1–4	155 (60.1)	148 (61.7)	303 (60.8)	
≥ 5	79 (30.6)	83 (34.6)	162 (32.5)	
Missing	24 (9.3)	9 (3.8)	33 (6.6)	
Marital status				< 0.001
Single/widowed/divorced/separated	72 (27.9)	41 (17.1)	113 (22.7)	
Married/Co-habitating	160 (62.0)	189 (78.8)	349 (70.1)	
Missing	26 (10.1)	10 (4.2)	36 (7.2)	
Education level				0.003
Less than high school/GED	78 (30.2)	50 (20.8)	128 (25.7)	
High school/GED	85 (33.0)	114 (47.5)	199 (40.0)	
Some college or more	68 (26.4)	62 (25.8)	130 (26.1)	
Missing	27 (10.5)	14 (5.8)	41 (8.2)	
Has a job/regular income				0.156
No	55 (21.3)	50 (20.8)	105 (21.1)	
Yes	176 (68.2)	176 (73.3)	352 (70.7)	
Missing	27 (10.5)	14 (5.8)	41 (8.2)	
Language spoken at home				0.004
Spanish	159 (61.6)	169 (70.4)	328 (65.9)	
English	18 (7.0)	5 (2.1)	23 (4.6)	
Spanish & English	54 (20.9)	56 (23.3)	110 (22.1)	
Q’anjob’al	1 (0.4)	0 (0.0)	1 (0.2)	
Missing	26 (10.1)	10 (4.2)	36 (7.2)	
Ability to speak English				0.001
Not at all	59 (22.9)	44 (18.3)	103 (20.7)	
Somewhat	108 (41.9)	139 (57.9)	247 (49.6)	
Very well	63 (24.4)	47 (19.6)	110 (22.1)	
Missing	28 (10.9)	10 (4.2)	38 (7.6)	

Just under half (48%; n = 240) reported having awareness of the public charge (Table 1). Among survey respondents, 22.1% indicated that they had not heard of any health programs or services available for adults and/or children in Oregon, and 39.2% indicated that they had never enrolled in or applied for these programs for themselves or their children (data not shown). Among survey respondents who had heard of the public charge rule (n = 240), 14.2% indicated that they were confident or very confident in their understanding of the rule (data not shown). Among those who had heard of the public charge, respondents most frequently got their information from television news, followed by social media, and friends, family, and coworkers (Table 2).

**Table 2:** Where survey respondents heard of the “Public Charge” rule, among respondents who reported being aware of the rule (n = 240). Percentages do not sum to 100% because respondents could select more than one information source

Information source	n (%)
Television news	170 (70.8)
Social media	78 (32.5)
Radio	38 (15.8)
Friends, family, coworkers	68 (28.3)
Community forum/Informational panel	10 (4.2)
Mexican consulate	7 (2.9)
Consultation with lawyer	29 (12.1)

**Fig. 1:**
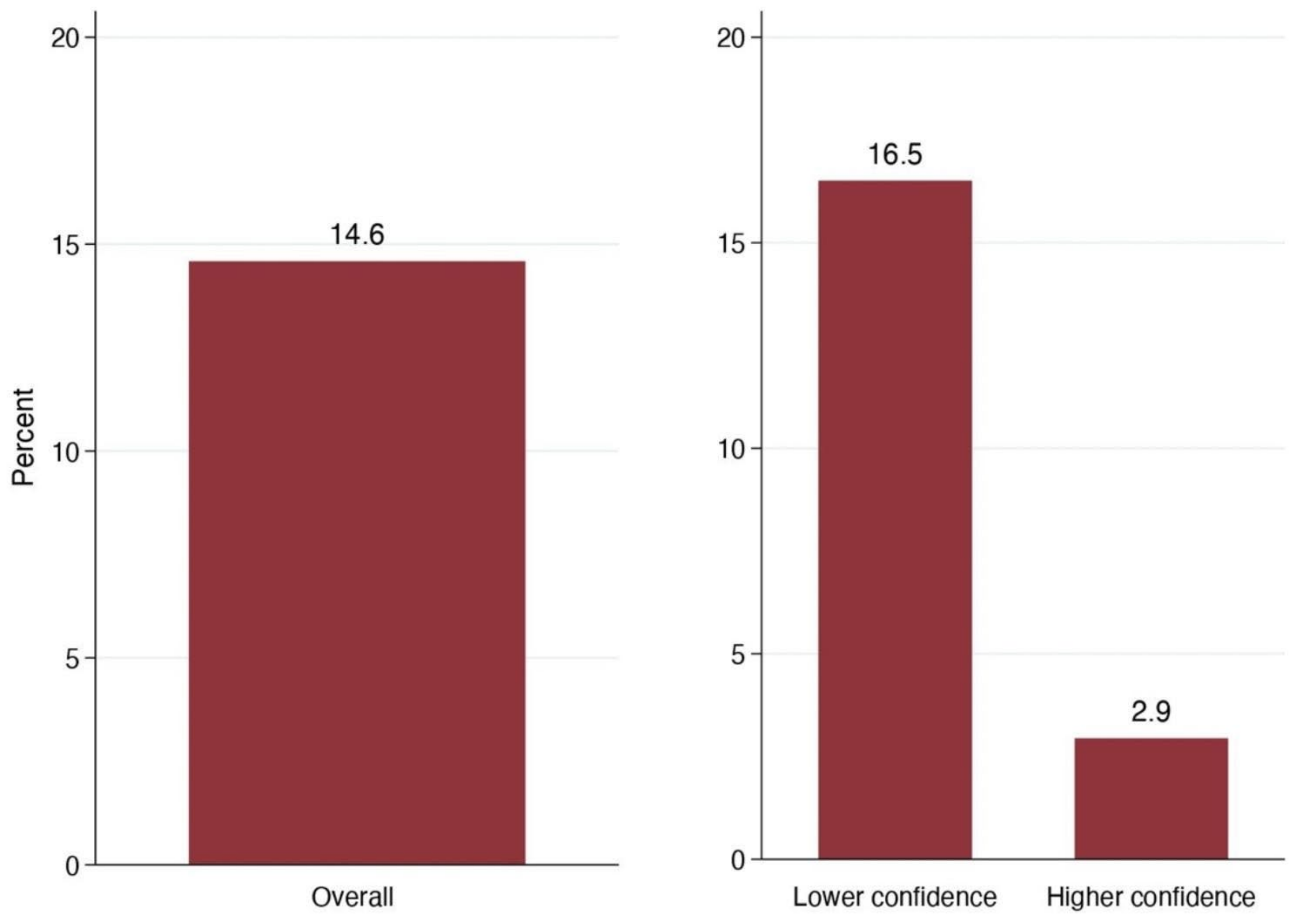
Percentage of survey respondents who had heard of the “Public Charge” rule (n = 240) that disenrolled themselves or a family member from a healthcare program due to concerns about the rule, overall (left) and by respondent’s confidence in their understanding of the rule (right). Bar labels indicate exact percentages

**Fig. 2:**
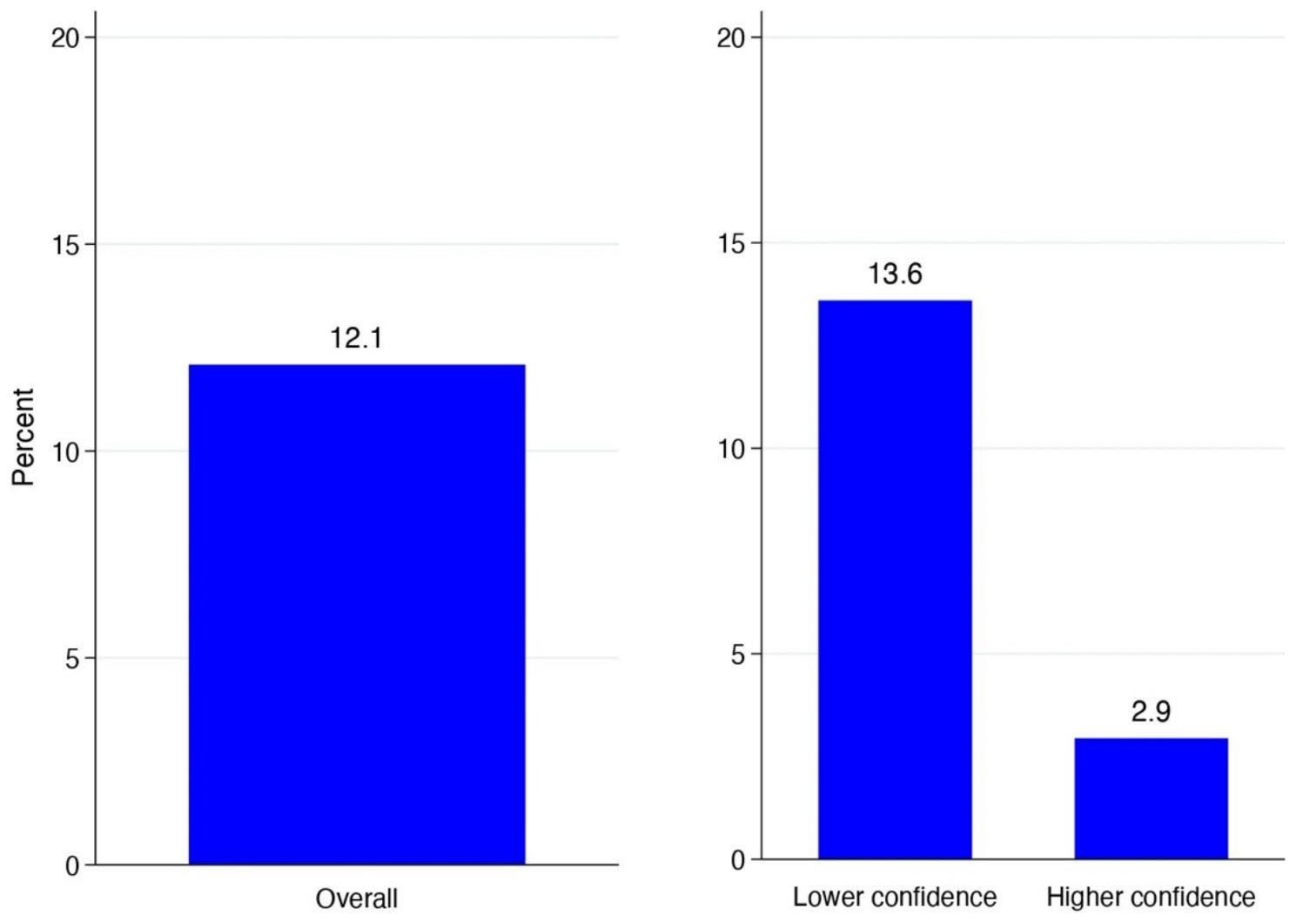
Percentage of survey respondents who had heard of the “Public Charge” rule (n = 240) that were hesitant to seek healthcare for themselves or a family member due to concerns about the rule, overall (left) and by respondent’s confidence in their understanding of the rule (right). Bar labels indicate exact percentages

Overall, 14.6% of respondents who had heard of the public charge had disenrolled themselves or family members from public healthcare programs (Fig. 1, **left**) and 12.1% were hesitant to seek care due to concerns about the public charge. (Fig. 2, **left).** However, both differed by the respondent’s confidence in their understanding of the rule: 16.5% of respondents with lower confidence in their understanding had disenrolled from some program compared with just 2.9% of respondents with higher confidence (Fig. 1, **right**). Similarly, 13.6% of respondents with lower confidence in their understanding were hesitant to seek care compared to 2.9% of those with higher confidence (Fig. 2, **right**).

In multivariable analyses, age and education were significantly associated with awareness of the public charge. An estimated 15.6% (95% CI 3.1–28.2%) of 18–24-year-olds knew about the public charge, compared with 42.6% (95% CI 29.3–55.9%) of 25–29-year-olds, 54.9% (95% CI 47.7–62.0%) of 30–39-year-olds and 49.5% (95% CI 43.0–56.0%) of respondents 40 and over (Table 3). Half (50.8%; 95% CI 45.5–56.0%) of respondents with a high school degree or GED or higher knew about the public charge, compared with 37.1% (95% CI 29.0-45.2%) of those with less than a high school education. See Table 3 for the full model and associated odds ratios.

**Table 3:** Adjusted odds ratios and probabilities (average marginal effects) of hearing about “Public Charge” rule from multivariable logistic regression model

Characteristics	Adjusted odds ratio			Average Marginal Effects	
	Estimate	95% CI	p-value	Estimate	95% CI
Age group					
18–24	0.14	0.05–0.38	< 0.001	15.6%	3.1–28.2%
25–29	0.59	0.31–1.13	0.113	42.6%	29.3–55.9%
30–39	Referent			54.9%	47.7–62.0%
≥ 40	0.79	0.52–1.21	0.280	49.5%	43.0–56.0%
Sex					
Female	1.39	0.92–2.11	0.115	50.8%	45.7–55.9%
Male	Referent			43.3%	35.9–50.8%
Education					
Less than high school/GED	Referent			37.1%	29.0-45.2%
High school/GED or more	1.87	1.19–2.95	0.007	50.8%	45.5–56.0%
Missing	5.48	0.92–32.65	0.062	72.1%	45.0-99.2%
Years in the US					
< 10	Referent			42.3%	32.1–52.5%
≥ 10	1.43	0.85–2.40	0.181	50.2%	45.2–55.3%
All my life	0.86	0.36–2.05	0.737	39.1%	23.3–54.8%
Missing	2.49	0.54–11.58	0.245	62.3%	32.6–92.1%
Relationship status					
Single/widowed/ divorced/separated	Referent			41.5%	31.9–51.1%
Married/Co-habitating	1.59	0.99–2.54	0.055	52.0%	46.4–57.6%
Missing	0.65	0.10–4.31	0.654	32.2%	0.0-67.8%
Ability to speak English					
Not at all	Referent			45.2%	35.1–55.4%
Somewhat/Very well	1.38	0.84–2.28	0.205	52.5%	46.8–58.3%
Missing	0.24	0.03–2.16	0.203	18.2%	0.0-45.8%

We identified four qualitative themes from the open-ended question about awareness of the public charge: barriers to citizenship (n = 63), right to access benefits (n = 8), details of programs in the public charge (n = 56) or feeling like a target (n = 27) (Table 4).

**Table 4:** Key themes about knowledge of the public charge

Theme	n	Definition	Exemplar quote
Barriers to citizenship	63	knowledge that a current or future change in legal status would be impacted by the public charge rule when obtaining aid from programs	“*Que las ayudas que uno recibe te perjudican a la hora de meter una aplicac[í]on con migracion”*. *“The help that one receives harms at the hour of submitting an application with immigration.”*
Rights to access benefits	8	individuals’ perception that they had a right to programs and/or their knowledge that not all public programs fall under the public charge	“N*o deber[í]a estar la carga publica porque hay personas que si necesita de esa ayuda, sobre todo ahorita en la pandemia.* *“There shouldn’t be a public charge because there are people who do need that help, especially right now in the pandemic.”*
Details of programs	56	knowledge of which programs fell under – or did not – the public charge, or described that they or their families would be a public charge if they used certain programs	*“Es cualquier programa de ayuda de gobierno para los inmigrantes como food stamps, welfare, Medicaid, etc”*. *“It is any government aid program for immigrant such as food stamps, welfare, Medicaid, etc.”*
Feeling like a target	27	the concept of being a target or feeling stereotyped by the government or society when using a public benefit because it would be seen as a negative action in society	“*Es una t[á]ctica anti-migratoria diri[g]ida hacia personas sin estado migratorio legal”* “*It is an anti-immigration tactic directed towards people without legal immigration status”*

## Discussion

The study purpose was to describe awareness of the public charge, confidence in knowledge about the public charge, and any disenrollment or avoidance of health service utilization due to the public charge among Oregon Latina/os. In addition, we identified factors associate with awareness of the public charge to inform outreach efforts. In our sample of Latina/os in Oregon, just under half (48%; n = 240) reported awareness of the public charge. Among individuals who reported awareness of the public charge, a minority (14.2%) had confidence in their understanding of the rule, 14.6% disenrolled themselves and/or children from healthcare programs, and 12.1% were hesitant to seek care due to concerns about the public charge; respondents with lower confidence in their understanding of the rule were more likely to disenroll and be hesitant to seek care than those with higher confidence. Qualitative results revealed four main themes (barriers to citizenship, rights to access benefits, details of programs, and feeling like a target) about awareness of the public charge.

We find that less than half of our sample was aware of the public charge, despite information available from state agencies and local non-profit organizations [22, 23]. These findings show that there is ongoing need for outreach and education about the public charge, perhaps with new modes of communication, focusing on places where our respondents reported getting information (e.g., television or social media) or in new spaces or trusted sources (e.g., the Mexican Consulate or schools). Our data suggest that outreach efforts among the local Latino community should target young people and those with less education, who were less likely to report awareness of the public charge in our study. Our qualitative data (theme: *details of programs)* provided some further insights into the misperceptions individuals have about which programs fall under the public charge and could be used to develop outreach materials.

Our results from Oregon support existing national evidence about use of public programs and the public charge modification under the Trump administration. In California, providers reported an increase in missed appointments among immigrant families, and 67% of immigrant parents reported concern about enrolling their children in Medi-Cal (California’s Medicaid program, similar to Oregon’s OHP) [24–26]. Reports from emergency department use in Texas by immigrants also support our findings about hesitancy to seek care and use health programs [27]. Furthermore, a majority of Latinos and Asians of diverse legal statuses in California reported negative perceptions and/or experiences related to the public charge rule [28]. A 2018 national survey reported that one in seven respondents (14%) avoided participating in public benefit programs in general (not only healthcare) due to “concerns about future green card status” [5]. This result is identical to our finding on disenrollment from public healthcare programs (also 14%). However, Oregon Medicaid covers noncitizen children up to age 19 using state funds [12, 13] and Oregon’s Reproductive Health Program covers non-citizen individuals for prenatal care and reproductive health services [14]; neither of these programs is part of the public charge. This finding supports the hypothesis of the “chilling effect” or “spillover effect” of the public charge policy.

Our findings should be interpreted with limitations in mind. First, our convenience sample was recruited at the Consulate General of Mexico in Oregon and may not be generalizable to all Mexican-origin Latinos in Oregon. Second, we were unable to directly ask about immigration status due to privacy concerns. However, we were interested in the “chilling effect” where all Latinos, regardless of immigration status, may be impacted by the public charge [29]. Third, we recruited Spanish and English-speaking participants only. One participant reported speaking an indigenous language at home (as well as speaking Spanish) but we were unable to offer our survey in indigenous languages. Finally, we did not use a priori conceptual framework for this exploratory study, but did base our study question and variables on previous literature. Study strengths include our community sample, recruited at the Mexican Consulate, which is used by US and foreign-born Latinos of Mexican origin of diverse immigration statuses regardless of education, income, or occupational status.

### Health equity implications

Policies that restrict access to public services and benefits based on immigration status are in direct conflict with health equity goals. Although the Biden administration reversed the Trump administration public charge modification, confusion about the public charge rule persists and the harm caused by the Trump administration’s modification may remain. This is important for equity now and in future generations because evidence shows that policies expanding eligibility or availability of insurance and public services for immigrants have a significant effect on their children’s access to care [30]. Our results suggest that ongoing outreach to the Mexican-origin community in Oregon is needed for the policy reversal to result in more uptake of public programs. We show that even in Oregon, a sanctuary state with state coverage for recent and undocumented immigrants where immigrants may feel safer than in more hostile states [31], concern about the public charge may negatively impact accessing public healthcare programs.

## Conclusion

Our study reveals limited awareness about the public charge rule among Mexican-origin Oregon Latino/as and that over one in seven (14.6%) reported disenrolling from public healthcare programs due to concerns about the public charge. Ongoing outreach is needed to ensure participation in public healthcare programs for the Mexican-origin Latino community in Oregon.

## Data Availability

The datasets used and/or analyzed during the current study are available from the corresponding author on reasonable request.
